# Decision-level adaptation in motion perception

**DOI:** 10.1098/rsos.150418

**Published:** 2015-12-02

**Authors:** George Mather, Rebecca J. Sharman

**Affiliations:** School of Psychology, College of Social Science, University of Lincoln, Lincoln LN6 7TS, UK

**Keywords:** motion adaptation, implied motion, response bias, normalization

## Abstract

Prolonged exposure to visual stimuli causes a bias in observers' responses to subsequent stimuli. Such adaptation-induced biases are usually explained in terms of changes in the relative activity of sensory neurons in the visual system which respond selectively to the properties of visual stimuli. However, the bias could also be due to a shift in the observer's criterion for selecting one response rather than the alternative; adaptation at the decision level of processing rather than the sensory level. We investigated whether adaptation to implied motion is best attributed to sensory-level or decision-level bias. Three experiments sought to isolate decision factors by changing the nature of the participants' task while keeping the sensory stimulus unchanged. Results showed that adaptation-induced bias in reported stimulus direction only occurred when the participants' task involved a directional judgement, and disappeared when adaptation was measured using a non-directional task (reporting where motion was present in the display, regardless of its direction). We conclude that adaptation to implied motion is due to decision-level bias, and that a propensity towards such biases may be widespread in sensory decision-making.

## 1. Introduction

Decision-making is a fundamental part of visual perception. We continually make decisions about the location, speed, direction, size, etc., of visual elements in the field of view during everyday tasks. Psychophysical research has established that these decisions are influenced by recent visual experience. Such adaptation effects manifest themselves under experimental conditions as a bias in the participant's responses to sensory stimuli. For example, an experiment on visual motion adaptation may measure a participant's ability to discriminate between leftwards and rightwards motion. Prior to adaptation responses usually show little or no bias in favour of either direction. However, after prolonged exposure to rightwards motion, responses are usually biased in favour of leftwards motion; a negative after-effect. Such biases can be explained by changes in the relative activity of neurons in the visual system that respond selectively to properties of visual stimuli [1,2]. For example, adaptation to rightwards motion desensitizes neurons that respond selectively to that direction, but leaves neurons selective for leftwards motion relatively unaffected, introducing a bias in subsequent activity levels which is manifest as a bias in observer responses.

Many electrophysiological studies of single-unit responses in primate cortex [3–5] and neuroimaging studies of the human cortex [6–8] have found evidence for changes in neural activity consistent with psychophysical motion adaptation effects. However, the psychophysical data cannot specify the source of the observer's response bias in adaptation experiments. Biases could be due to adaptation in sensory neurons or to shifts in the observer's criterion for selecting one response rather than the alternative [9–11]. Thus, sources of bias in adaptation experiments can be divided into two classes. We define *sensory-level adaptation* (SLA) as an adaptation-induced bias in the pattern of neural responses (as described above), which leads to a response bias, and *decision-level adaptation* (DLA) as a response bias induced by a change in the way that neural responses are interpreted in order to reach a decision. For example, the visual system may maintain an unbiased criterion for deciding between ‘left’ and ‘right’ on the basis of experience that the two directions occur equally often in visual images. Motion adaptation experiments introduce a bias in the pattern of stimulation in favour of one direction which the visual system may attempt to neutralize by introducing a compensatory decision bias in its criterion (in favour of the opposite direction). This kind of bias would amount to recalibration or error-correction which re-labels the lines coming from stimulus-tuned neurons to ‘normalize’ skewed neural response distributions even in the absence of sensory shifts [12–16]. However, other kinds of bias could also contribute to DLA [17]. Evidence to date indicates that DLA is relatively weak. In a psychophysical study of motion adaptation, for example, Morgan *et al.* [16] found some evidence for recalibration though their adaptation effect was predominantly attributable to SLA.

Psychophysical studies of adaptation-induced response bias frequently confound SLA and DLA, because they cannot specify the source of the bias [9,11,17]. For example, Winawer *et al.* [18] showed observers a sequence of static adapting images which all depicted ‘implied motion’; photographs of human figures engaged in movement (walking, running and jumping) in a consistent direction. Following this adaptation, observers were shown dynamic test stimuli containing dots which actually moved either in the same direction as depicted in the adapting images or in the opposite direction. Results showed a slight shift in observers' sensitivity to the test motion, favouring stimuli moving in the opposite direction to adaptation. This adaptation effect is surprising because cortical neurons selective for motion do not respond to stationary patterns [19]. Nevertheless, Winawer *et al.* [18] attributed the response bias to SLA, arguing that the adaptation was due to excitation of motion-selective neurons by form-coding neurons responsive to implied motion. However, the relatively small effect of implied motion adaptation may be due to some form of DLA: a slight shift in the criterion for ‘left’ versus ‘right’ responses induced by inspection of directional implied motion. Such an involvement of higher level decisional processes is consistent with a recent report [20] that visual awareness is necessary for implied motion to produce adaptation, whereas it is not necessary to produce adaptation to low-level motion [21].

This paper reports a series of three psychophysical experiments designed to assess whether implied motion adaptation is best explained by SLA or DLA. We sought to isolate decision factors by changing the nature of the task performed by the participants while keeping the sensory stimulus unchanged. If the adaptation effect is due to SLA, then it should not be affected by changes to the task, but if it is due to DLA then it should be affected by changes to the task. In the first experiment, we established a baseline measure of adaptation to implied motion using a standard procedure [18,20], against which we could compare later results.

## 2. Experiment 1

### 2.1 Methods

#### 2.1.1 Participants

Eight participants took part, two males and six females, with normal or corrected-to-normal vision. Mean age was 31, with a range of 25–59 years old. All except two participants (the authors) were naive as to the purpose of the experiment. All participants gave their informed consent to participate in the study.

#### 2.1.2 Apparatus

A ViewPixx 3D lite (VPixx, Quebec, Canada) display system was used for stimulus presentation. The display had a resolution of 1920×1080 pixels, a refresh rate of 120 Hz and a screen width of 52 cm. The system was gamma-corrected using a LS100 luminance meter (Minolta, Osaka, Japan). A chin rest was used to ensure that the participant viewed the display from a constant distance of 300 cm (0.0052 deg arc per image pixel). Stimulus presentation and data collection were controlled by PsychoPy [22] running on a Dell desktop PC.

#### 2.1.3 Adapting stimuli

Implied motion images were individual video frames taken from a recording of the London Marathon 2014 made using a GZ-GX1BEK camera (JVC, Yokohama, Japan). The camera was fixed in place to one side of the route, with a view orthogonal to the path of the runners as they passed by from left to right. The recording was made at 125 frames per second at a resolution of 720×576 pixels. To prepare the stimulus set the recording was de-interlaced and the individual frames were extracted. Each frame was converted to greyscale and cropped to the central 512×512 pixels. A small number of frames were excluded from the stimulus set because they did not contain any running figures, contained figures that were not moving, or moving in the opposite direction to the marathon runners. The resulting stimulus set contained 20 513 frames. These frames were presented either as recorded to depict rightwards implied motion or were flipped horizontally to create leftwards implied motion. Each image frame subtended 3.0×3.0 deg arc and was presented against a uniform grey background of 53.78 cd m^−2^ with a central fixation point.

#### 2.1.4 Test stimuli

The test display consisted of 100 dots each with a diameter of 10 pixels (3.12 min arc) randomly positioned within a square of width 3 deg arc. The frame-to-frame *y*-displacement of each dot was randomly drawn from a Gaussian probability density function with a mean of zero and standard deviation of 10 pixels (3.12 min arc). The frame-to-frame *x*-displacement was also randomly drawn from a Gaussian distribution, with a mean of *x* pixels and standard deviation of 10 pixels (3.12 min arc). The value of *x* varied from trial to trial under the control of an adaptive psychophysical procedure which converged on the *x*-displacement required for the participant to reach a specified level of performance in the task (see below). If the value of *x* was zero, dots each took a random walk across the screen with no coherent directional signal; if the value of *x* was below zero each dot tended to move in a leftward direction and if the value was above zero each dot tended to move in a rightward direction. A similar stimulus has been used previously to measure adaptation-induced biases in reported stimulus direction [23].

#### 2.1.5 Procedure

During the adaptation period, a random selection of the implied motion images was presented at a rate of five images per second for 60 s. Then followed a test/top-up cycle in which the test stimulus appeared for 250 ms, followed by a 4 s period of top-up adaptation (containing a new random selection of images). Each adapting stimulus was shown in a separate experimental session. In half of the sessions, the adapting stimuli implied rightwards motion, and in the other half of sessions the adapting stimuli implied leftwards motion. The running order of adapting conditions and adapting directions was randomized across participants. Within a session, half of the test presentations (randomly selected) contained leftward dot motion and the rest contained rightward motion. The participant was instructed to report whether the test stimulus contained leftward or rightward motion by pressing one of two corresponding response keys with the thumb of either their left hand or their right hand (ResponsePixx, VPixx).

A QUEST adaptive psychophysical staircase procedure was used to measure each participant's threshold for correctly reporting the direction of test stimuli [24]. QUEST provides a trial-by-trial estimate of the most probable threshold value which guides the stimulus value presented on each successive trial in order to converge on the threshold. The test *x*-displacement (mean of the Gaussian probability density function) presented on each trial was based on the participant's performance in previous trials in order to estimate the leftwards (or rightwards) displacement required for the participant to correctly identify the test direction in 82% of trials. Two separate randomly interleaved staircases were run in each session to separately measure thresholds for rightwards and leftwards motion, with 40 trials in each staircase (the staircase estimating rightwards thresholds only selected rightwards displacements; similarly, the leftwards staircase only selected leftwards displacements). The participant's threshold for each direction was taken as the final QUEST estimate following the last trial of the relevant staircase. Preliminary observations had established that this procedure was an appropriate method of measuring small adaptation-induced biases in each direction.

### 2.2 Results and discussion

The aim of the experimental procedure was to measure a response bias on the part of the participant which favoured one of the two test directions, whether due to SLA or DLA. In the absence of any bias, the obtained displacement threshold for reporting leftward motion should be equal to the threshold for reporting rightward motion (disregarding the sign of displacement). On the other hand, if the participant had a bias in favour of, say, reporting rightward motion, then the measured threshold for rightward motion would be lower than that for leftward motion, because a smaller rightward displacement would be required to reach the 82% threshold. The participant's tendency to give a rightwards response would reduce the amount of physical rightwards displacement required to reach threshold. By contrast, more leftward displacement would be required to overcome the rightward response bias and reach the threshold for leftward motion. So our analyses focus on comparisons between rightward and leftward thresholds in different adapting conditions.

**Figure 1.**
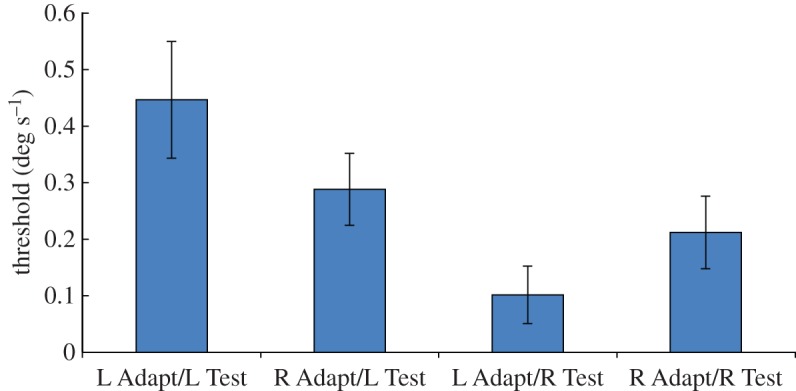
Results of Experiment 1. Mean displacement threshold for correctly reporting the direction of leftward and rightward dynamic random dot test stimuli (‘L Test’ and ‘R Test’, respectively), following adaptation to static left-facing or right-facing implied motion images (‘L Adapt’ and ‘R Adapt’, respectively). Vertical bars indicate±1 s.e.m. Test dots were randomly distributed in the entire stimulus aperture.

Figure 1 shows the mean thresholds for leftward and rightward test stimuli (final QUEST estimates) after adaptation to either left- or right-facing implied motion images. Notice that both thresholds for leftward test stimuli (two left-hand columns) are slightly higher than those for corresponding rightward test stimuli (two right-hand columns), indicating that overall participants had a rightwards response bias.

In order to establish whether adaptation to the direction of implied motion affected subsequent perception of physical motion, we must consider the interaction between adapting and test directions. Figure 1 shows that the mean threshold for leftward test stimuli was lower after adapting to right-facing implied motion than after adapting to left-facing implied motion. Conversely, the threshold for rightward test stimuli was lower after adapting to left-facing implied motion than after adapting to right-facing implied motion. This interaction between adapting and test directions is consistent with the specific prediction of implied motion adaptation [18]; thresholds are lower when the test and adaptation directions differ than when they are the same. A one-tailed two-factor repeated measures ANOVA revealed a significant main effect of test direction [*F*_1,7_=4.28; *p*=0.039] and a significant interaction between adapting and test directions [*F*_1,7_=4.47; *p*=0.036]. The main effect of test direction confirms the overall bias of participants in favour of rightwards responses, and the interaction between adapting and test directions confirms the presence of a direction-selective adaptation effect.

## 3. Experiment 2

Biases of the kind found in Experiment 1 could be introduced either at the sensory level or at the decision level. Experiment 2 tested whether removing the directional component of the participant's task affected the outcome. If the basis for the measured adaptation effect is sensory, it should not be affected by a change in the task. However, if the bias is due to a shift in the criterion for reporting ‘left’ versus ‘right’ then it should disappear in a non-directional task.

### 3.1 Methods

All methodological details were identical to Experiment 1, including the same observers, with the following exceptions. In each test presentation, only the upper or the lower half of the stimulus display (randomly selected) contained a directional component; the Gaussian distribution from which dot *x*-displacements were randomly drawn had a non-zero mean. In the other half of the stimulus display, the means of both the *x*- and *y*-displacement distributions were zero, creating incoherent random motion. In half of the test trials (randomly selected), the dot motion was leftwards and in the other half it was rightwards.

The participant was required to press one of two response keys to indicate which half of the display contained more coherent motion (top or bottom), regardless of its direction. Randomly interleaved adaptive staircases were run for leftward and rightward test trials, in order to find the displacement in each direction required for the participant to correctly report the half of the stimulus which contained motion.

Experiments 1 and 2 were planned as a pair, so all the sessions relating to them were run in a different random order for each participant.

### 3.2 Results and discussion

**Figure 2.**
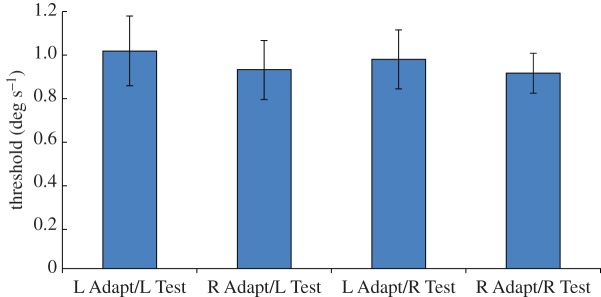
Results of Experiment 2. Only one half of the stimulus aperture, either upper or lower, contained coherently moving dots; the other half contained incoherently moving dots. In half of the test presentations, the coherent motion was leftward, and in the remainder it was rightward. Participants were instructed to report which half of the display contained coherent motion, regardless of its direction. The graph plots the mean displacement threshold for correctly reporting the half of the display containing coherent motion, when the motion was leftward or rightward (‘L Test’ and ‘R Test’, respectively), following adaptation to static left-facing or right-facing implied motion images (‘L Adapt’ and ‘R Adapt’, respectively). Vertical bars indicate±1 s.e.m.

Figure 2 summarizes the results of Experiment 2, using the same layout as figure 1. As in Experiment 1, we ran a one-tailed two-factor repeated measures ANOVA on the data. In contrast to Experiment 1, statistical analysis shows no significant differences [Test direction: *F*_1,7_=0.14; *p*=0.72; Adapt×Test:*F*_1,7_=0.012; *p*=0.46].

The change in the task appears to have removed the bias from the participant's responses, indicating that decision factors are responsible for the bias found in Experiment 1. However, it is clear from the higher threshold values that the task is more difficult than that in Experiment 1, presumably because the stimulus contains only half the number of coherently moving dots. This reduction in signal strength may also have made it more difficult to measure a sensory adaptation effect.

## 4. Experiment 3

Experiment 3 was designed to have the same decision factors as Experiment 1 (a directional response), but the same stimulus arrangement as Experiment 2 (a directional signal in only half of the display). If the adaptation effect is due to decision factors, then it should re-appear in Experiment 3.

### 4.1 Methods

Stimulus details were identical to those in Experiment 2, with only half of the test display containing coherent motion, but the participant's task was the same as in Experiment 1. Participants were asked to press one of two response keys to indicate the dominant direction of motion they saw in the test pattern, regardless of whether it appeared in the top or bottom half of the display. The adaptive staircases converged on the *x*-displacement required for participants to attain 82% correct performance.

Experiment 3 was devised after Experiments 1 and 2, so its sessions were all run after those for the earlier experiments. We employed the same participants to ensure comparability in the data, with session order randomized across participants.

### 4.2 Results and discussion

**Figure 3.**
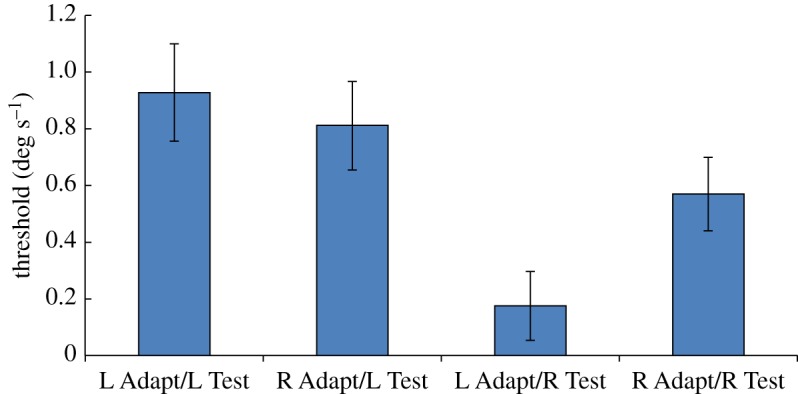
Results of Experiment 3. Stimuli were identical to those in Experiment 2, but the task was the same as that in Experiment 1. Only one half of the test stimulus aperture, either upper or lower, contained coherently moving dots; the other half contained incoherently moving dots. In half of the test presentations, the coherent motion was leftward, and in the remainder it was rightward. Participants were instructed to report the direction of coherent motion in the test pattern, regardless of where in the display it appeared. The graph plots the mean displacement threshold for correctly reporting the direction of coherent motion, when the motion was leftward or rightward (‘L Test’ and ‘R Test’, respectively), following adaptation to static left-facing or right-facing implied motion images (‘L Adapt’ and ‘R Adapt’, respectively). Vertical bars indicate±1 s.e.m.

The results plotted in figure 3 show that the adaptation effect re-appeared in Experiment 3. The pattern of results is very similar to that in Experiment 1 although threshold values are higher overall (similar to Experiment 2). As in Experiments 1 and 2, we ran a one-tailed two-factor repeated measures ANOVA on the data. There were significantly higher thresholds for leftward test stimuli [*F*_1,7_=4.54; *p*=0.036], and a significant interaction between adapting and test directions [*F*_1,7_=5.21; *p*=0.028].

Even though identical stimuli were presented in Experiments 2 and 3, the outcomes were quite different. When the task involved a directional judgement (Experiment 3), the results showed a bias consistent with an adaptation effect, but when the task did not involve a directional judgement (Experiment 2) no such bias was evident.

## 5. General discussion

These results indicate that the implied motion adaptation effect is due to changes in decision factors rather than in sensory factors. The direction of the effect indicates that after viewing an image containing rightward information, participants have a slight bias favouring leftwards decisions (and a corresponding bias towards the right after viewing leftward images). This bias is consistent with decision-level normalization or recalibration: the visual system has no independent way of verifying whether transitory biases in sensory messages are due to stimulus bias or to moment-to-moment fluctuations in neural responsiveness. Calibration of sensory messages must therefore be based on experience of the visual world. Averaged over a long period, leftwards and rightwards motion judgements are equally likely to occur. So the null point or norm between left and right should be unbiased and correspond to the average of the left–right dimension over the recent past. Our results indicate that selective exposure to one direction, either real or implied, biases the norm and shifts the null point towards the new norm. After exposure to ‘right’, the neutral point between ‘left’ and ‘right’ responses shifts towards the right, so that weak rightwards stimuli are classified as leftward; a left bias. This is different from SLA because it is due to a shift in decision criterion, not a change in the sensory signal itself. Our adaptation effect is consistent with such a decision-level recalibration process, but we cannot rule out other possible characterizations of the decision bias. For example, one could argue that the shift is due to expectations. If participants were familiar with sensory adaptation studies they may have harboured an expectation that after viewing rightwards motion they should see leftwards motion more often.

The decision-level normalization hypothesis assumes that prior to adaptation responses are not biased in favour of one direction or the other. However, the results of Experiments 1 and 3 show a slight overall bias in favour of rightwards responses (we also ran control sessions in which the adapting stimulus was a grey screen, and thresholds showed the same bias in favour of rightwards responses). This is unlikely to reflect a sensory imbalance, because it was not evident in Experiment 2, and may reflect a relatively high-level bias towards the right. Palmer *et al.* [25] found that observers have an aesthetic preference for right-facing objects in pictures, and more frequently take photographs of objects facing right when given the choice. Similarly, Walker [26] searched Google Images and found that right-facing implied motion images are significantly more common than left-facing images. Both authors speculate that the right-facing bias could be due to either left-to-right reading direction in English, the common use of a spatial metaphor for time running from left to right, hemispheric processing, or handedness (all but one of our participants reported that they are right-handed). These high-level biases would be consistent with the obtained predisposition on the part of our participants in favour of rightwards decisions.

Many psychophysical experiments on motion adaptation employ directional judgements. When studying perceived direction it seems natural for the researcher to ask participants to report the direction that they perceive in the stimulus. However, while there are undoubtedly significant sensory factors that contribute to biases in participants' directional responses, our results support the view that a component of any bias measured using directional tasks may come from DLA. For example, Pavan *et al.* [27] reported a directional shift in apparent position following adaptation to implied motion. Decision-level effects could have contributed to this result given that the participants' task was a directional judgement (‘Was the top blob to the left or right of the bottom blob?’). It is possible that corresponding biases exist in other sensory dimensions, particularly those with null points. For example, in the case of red versus green colour after-effects, one component of the red versus green judgement may be due to decision-level recalibration. If so, then a task that does not explicitly require a colour judgement, such as an odd-one-out task, may produce different results and a purer measure of sensory adaptation. The adaptation effect found in Experiments 1 and 3 was quite small, as was that reported by Winawer *et al.* [18]. Larger sensory effects might swamp decision factors, making decision factors important particularly in situations where the sensory data are quite noisy and ambiguous.

Traditionally experimenters have tended to view decision effects as artefacts but we would argue that they are an integral part of perceptual judgements, whether inside the laboratory or outside, and are therefore worthy of further investigation. For example, decisions in sports events largely rely on noisy or ambiguous perceptual data, and the frequent disagreements between players, spectators and officials may be partly due to decisional factors such as previous experience of the sport or particular game, and knowledge about the participants. On-the-spot decisions about fouls are particularly difficult to make on the basis of sensory evidence alone given the speed of modern sport, and are therefore likely to be affected by decision bias and normalization effects. A study of foul calls in US professional basketball games found that officials are more likely to call fouls in favour of the team with the fewest fouls, so as to even out the number of fouls on each side during the game [28]. Similarly, analysis of penalty awards in German professional football games shows that the award of a first penalty to one team raises the referee's evidence criterion for the same team but lowers it for the opposing team [29]. In both cases, it appears that officials shift their neutral point on the assumption that fouls and penalties even out over time.

To conclude, in experiments using identical stimuli we found that biases in response induced by adaptation to directional implied motion only occurred when the experimental task required a decision about stimulus direction. Data suggest that the implied motion adaptation effect is due to changes in decision factors rather than sensory factors. These results are consistent with decision-level normalization or recalibration of sensory messages, which may be widespread in sensory decision-making.

## Data accessibility

Archived data are available at Dryad: http://dx.doi.org/10.5061/dryad.26v53.
